# Tropical Medicine

**Published:** 1916-09-02

**Authors:** 


### TROPICAL MEDICINE.

**London School of Tropical Medicine.**—This is one of the few post-graduate schools which, though affected in one way by the war, has maintained the full course of its curriculum uninterrupted during the last twelve months. On an average, nearly 200 students enter annually, but during the three sessions which have been held in the last twelve months the total number has fallen to thirty. This falling-off is principally occasioned by the fact that very few officers of the Colonial Medical Service have been seconded for study; the same applies to officers in the Indian Medical Service and Royal Navy, and the number of missionary students attending has been less. The war has robbed the school of the services of the Helminthologist, Protozoologist, and some of the demonstrators, but the honorary medical staff, teachers, and lecturers are still carrying on the educational work of the establishment. The only increase has been in the number of women students.

There is no diminution in the number of tropical cases admitted to the wards; indeed, there has been rather an increase, for officers suffering from tropical disease sent home from Persia, Egypt, and the Dardanelles are admitted for treatment under an arrangement with the War Office.

Though the reduced number of students has materially affected the financial position of the School, there have been many economies effected which have reduced the deficit to a minimum. The School offers to receive Belgian graduates and to afford them free tuition, board and residence, and already four Belgian practitioners have availed themselves of the hospitality of the School. The next session will open on October 2.

**Liverpool School of Tropical Medicine.**—The Liverpool School of Tropical Medicine is carried on in conjunction with the University of Liverpool, the Royal Southern Hospital, and the Royal Infirmary, for the purposes of research and clinical teaching respectively. Special wards at the hospitals have been set apart for the reception and treatment of tropical diseases, and facilities for investigation and study are given in the Johnston Laboratories of the University. The fee for a full course (lasting thirteen weeks) is 13 guineas. A short course, specially arranged for advanced instruction, is also held during June (fee 4 guineas).

**Both Cambridge University and the English Conjoint Board** hold examinations for the Diploma in Tropical Medicine. Neither of these bodies teaches Tropical Medicine; candidates derive their knowledge of the subject elsewhere, usually at either the London or the Liverpool School.

**Edinburgh Diploma of Tropical Medicine.** The University of Edinburgh grants a Diploma in Tropical Medicine and Hygiene. The examinations are held in December and June. The fees for the first and any subsequent appearance are: Practical bacteriology, £1 1s.; diseases of tropical climates, £1 1s.; tropical hygiene, £1 1s.; tropical clinical medicine, £1 1s.; total, £4 4s.

